# Surveillance and laboratory detection for non-polio enteroviruses in the European Union/European Economic Area, 2016

**DOI:** 10.2807/1560-7917.ES.2017.22.45.16-00807

**Published:** 2017-11-09

**Authors:** Heli Harvala, Aftab Jasir, Pasi Penttinen, Lucia Pastore Celentano, Donato Greco, Eeva Broberg

**Affiliations:** 1 Public Health Agency of Sweden, Stockholm, Sweden; 2 European Programme for Public Health Microbiology Training (EUPHEM), European Centre for Disease Prevention and Control (ECDC), Stockholm, Sweden; 3 European Centre for Disease Prevention and Control (ECDC), Stockholm, Sweden

**Keywords:** Europe, viral infections, respiratory infections, viral encephalitis, viral meningitis, laboratory surveillance, laboratory methods, molecular typing

## Abstract

Enteroviruses (EVs) cause severe outbreaks of respiratory and neurological disease as illustrated by EV-D68 and EV-A71 outbreaks, respectively. We have mapped European laboratory capacity for identification and characterisation of non-polio EVs to improve preparedness to respond to (re)-emerging EVs linked to severe disease. An online questionnaire on non-polio EV surveillance and laboratory detection was submitted to all 30 European Union (EU)/European Economic Area (EEA) countries. Twenty-nine countries responded; 26 conducted laboratory-based non-polio EV surveillance, and 24 included neurological infections in their surveillance. Eleven countries have established specific surveillance for EV-D68 via sentinel influenza surveillance (n = 7), typing EV-positive respiratory samples (n = 10) and/or acute flaccid paralysis surveillance (n = 5). Of 26 countries performing non-polio EV characterisation/typing, 10 further characterised culture-positive EV isolates, whereas the remainder typed PCR-positive but culture-negative samples. Although 19 countries have introduced sequence-based EV typing, seven still rely entirely on virus isolation. Based on 2015 data, six countries typed over 300 specimens mostly by sequencing, whereas 11 countries characterised under 50 EV-positive samples. EV surveillance activity varied between EU/EEA countries, and did not always specifically target patients with neurological and/or respiratory infections. Introduction of sequence-based typing methods is needed throughout the EU/EEA to enhance laboratory capacity for the detection of EVs.

## Introduction

A total of 116 enterovirus (EV) types have been identified from humans, and of these, 45 have been discovered in the past 10 years [1]. EVs include polioviruses (PV), coxsackie A viruses (CAV), coxsackie B viruses (CBV), echoviruses (E) and numbered EVs. Based on a molecular classification, this diverse group of RNA viruses are divided into four EV species (EV-A to EV-D) [2-4]. Rhinoviruses are genetically closely related to EVs, forming three further species within the *Enterovirus* genus (HRV-A to HRV-C) [2,3].

EVs cause a wide spectrum of infections in humans, including non-specific febrile illness and viral exanthema, respiratory infections, hand, foot and mouth disease (HFMD), myocarditis, meningitis, encephalitis and, rarely, acute flaccid paralysis (AFP) [5]. Species A EVs are known for their ability to cause HFMD, and EV-A71 has also been associated with geographically widespread outbreaks of neurological infections, mainly in the Asia Pacific region [6]. Circulation of EV-A71 has also been documented in Europe [7-13]. Species B EVs are the main causes of aseptic meningitis in Europe [14-16]. Clusters of respiratory disease caused by EV-D68, a species D EV, occasionally leading to severe neurological complications, have previously been reported in Europe and North America [17-22].

The classical method to diagnose EV infection has been virus isolation by cell culture from clinical specimens, followed by neutralisation assay to determine the serotype [23]. Although more efficient molecular detection techniques have mostly replaced these slow and laborious cell culture methods in primary diagnostic laboratories, virus isolation still plays a crucial role in polio surveillance and all polio cases are confirmed by in vitro cell culture [24-26].

Reverse transcriptase polymerase chain reactions (RT-PCR) used for diagnosing EV infections usually target the 5’ untranslated genomic region (5’UTR), which is highly conserved within the *Enterovirus* genus and therefore enables detection of most 116 EV types within one assay with comparable sensitivity [27]. Some of the PCR assays used are for specific detection of EVs only, but some assays detect both EVs and rhinoviruses. However, some of the newly identified EV types within species C (e.g. EV-C104) possess a genetically divergent 5’UTR and are hence undetectable by most PCR methods [28,29]. Amplification and (partial) sequencing of a structural gene region such as VP1 are required for reliable EV type identification [30].

Although EV surveillance is mainly recommended by the World Health Organization (WHO) as a component of the Global Poliovirus Elimination Action Plan, it also provides parallel data on non-polio EV detection. The main principal public health needs for non-polio EV surveillance include outbreak detection and response, and monitoring of EV types associated with severe disease [26]. However, as current EV surveillance primarily aims for identification of poliovirus, detection of EV types typically targets clinical cases with symptoms of AFP, meningitis and encephalitis as well as occasional cases with gastrointestinal or respiratory symptoms [26].

Polioviruses, like most other EVs, are transmitted mostly via the faecal–oral route [5]. Polioviruses can be isolated from faeces for several weeks after onset of symptoms, from throat secretions in the first 2 weeks of illness and occasionally from cerebrospinal fluid (CSF) [5]. Therefore, stool samples are typically used for poliovirus surveillance. The limitations of current laboratory-based EV surveillance for the identification of non-polio EV became apparent when the first EV-D68 cluster was detected in Europe in 2010 [20]. Existing diagnostic screening of stool samples together with occasional CSF samples was ineffective for the detection of EV-D68 as this EV type, which primarily causes respiratory infections, is only very rarely detected in stool or CSF samples [21,22].

To our knowledge, there are no previous Europe-wide data available on the methods currently used for EV surveillance, detection and typing. The main aim of this study was to record European capability for identification and characterisation of non-polio EVs in order to improve the laboratory response to (re)-emerging EVs linked to severe disease.

## Methods

An online questionnaire on non-polio EV surveillance and laboratory detection was submitted to all European Union (EU)/European Economic Area (EEA) countries via the National Coordinators of the European Centre for Disease Prevention and Control (ECDC) Coordinating Competent Bodies. Questionnaires were also sent to the Operational Contact Points for Influenza Surveillance as well as to the National Focal Points for Influenza, Microbiology and Vaccine Preventable Diseases. The National Coordinators were asked to assign a person or persons from their country to fill in the survey from the national perspective. Specific questions were asked about the laboratory methods used for detection and typing/characterisation of non-polio EVs and it was recommended that a person with detailed understanding of laboratory methods should respond to the questionnaire. Two reminders were sent to the National Coordinators and Operational Contact Points. Only one survey response per country was considered in the final analysis; data obtained from multiple responses were merged into a single country-specific response. The questionnaire was sent on 11 April 2016, and the responses were requested by 30 April 2016.

The questionnaire was divided into four parts. The first part recorded the respondents’ background information and the second part focused on the current non-polio EV surveillance systems in place. Information was collected on whether the country’s EV surveillance included identification of poliovirus circulation via typing/characterisation of EV-positive stool samples, and identification of poliovirus and other EV via typing/characterisation of variety of clinical EV-positive specimens, and whether the country had reporting and/or surveillance systems for HFMD and EV-D68. Furthermore, we also specifically asked whether surveillance for EV-D68 was performed via: (i) sentinel surveillance of influenza-like illness (ILI) and/or other respiratory virus infections; (ii) AFP surveillance; (iii) EV surveillance including respiratory samples; (iv) laboratory reporting of EV-D68 positive samples or (v) in another way. The third part was on laboratory methods used for detection of non-polio EV, and the fourth on further typing/characterisation of EV-positive specimens. Countries were also asked to estimate how many EV-positive samples were referred for typing/characterisation annually, and to provide exact numbers for 2015 if available. We also calculated the proportion of EV-positive samples typed per 100,000 inhabitants for the countries that were able to provide these data. If the country was unable to provide an exact number, we used the upper limit of estimation for the calculations.

Data on primers and probes used for non-polio EV detection were collected either directly from the survey answers, and/or from the references provided by responders. These were compared with the published sequences of all known EV types to identify potential mismatches. The alignment was performed in Simple Sequence Editor (SSE) version 1.2 [31].

## Results

A total of 29/30 EU/EEA countries responded to the survey (97% response). The majority of respondents (n=27) were microbiologists, and several of these (n=7) were additionally trained in epidemiology. Most respondents worked at their national EV laboratory (n = 25), and some were also associated with the influenza and other respiratory viruses reference laboratory (n = 9).

### Surveillance systems in place

The properties of national surveillance systems and laboratory methods used for non-polio EVs in 29 EU/EEA countries are shown in Table.

**Table:** Properties of national surveillance systems and laboratory methods used for non-polio enteroviruses, European Union/European Economic Area, 2016 (n=29 countries)

Properties	Number of countries/ Number of countries responding
**Type of surveillance system**	
EV surveillance via typing of clinical EV-positive samples	26/29
Surveillance includes neurological infections	24/26
Surveillance includes respiratory infections	16/24
Surveillance includes HFMD	18/23
Surveillance includes myocarditis	17/23
Surveillance includes haemorrhagic conjunctivitis	14/23
Surveillance includes post-mortem investigations	15/23
Reporting system for EV-D68	11/28
Surveillance for EV-D68	11/29
Reporting system for HFMD	4/28
Surveillance for HFMD	2/28
General features of EV testing	
National recommendations for EV testing	11/29
Collect CSF, respiratory and faecal sample if AFP suspected	3/26
Collect CSF, respiratory and faecal sample if neurological infection	5/26
Would recommend collection of vesicle swab if HFMD suspected	18/22
Test all CSF samples for EVs	8/17
Test all respiratory samples for EVs	3/17
National laboratory offers any EV testing	28/28
National laboratory offers primary EV testing	25/29
Non-national laboratories offer also primary EV testing	23/27
Non-national laboratories offer also EV typing	11/26
National laboratory participates into external QA on detection	25/29
National laboratory participates into external QA on characterisation/typing	20/28
National laboratory capability for EV identification	
Virus isolation	27/29
Any molecular method	28/29
Gel-based RT-PCR	11/29
Real-time RT-PCR	16/29
EV-D68 specific real-time RT-PCR	7/28
Commercial methods	10/29
IgG and/or IgM serology	8/28
National laboratory capability for EV characterisation	
Any EV characterisation	26/29
Typing only by neutralisation test	7/29
Typing only by sequence analysis	19/29
Typing by neutralisation test and sequence analysis	9/29
Only culture-positive EVs typed	10/29
Non-cultured EV-positive samples typed by sequence analysis	16/29

AFP: acute flaccid paralysis; CSF: cerebrospinal fluid; EV: enterovirus; HFMD: hand, foot and mouth disease; QA: quality assurance; RT-PCR: reverse transcription-polymerase chain reaction.

**Figure 1:**
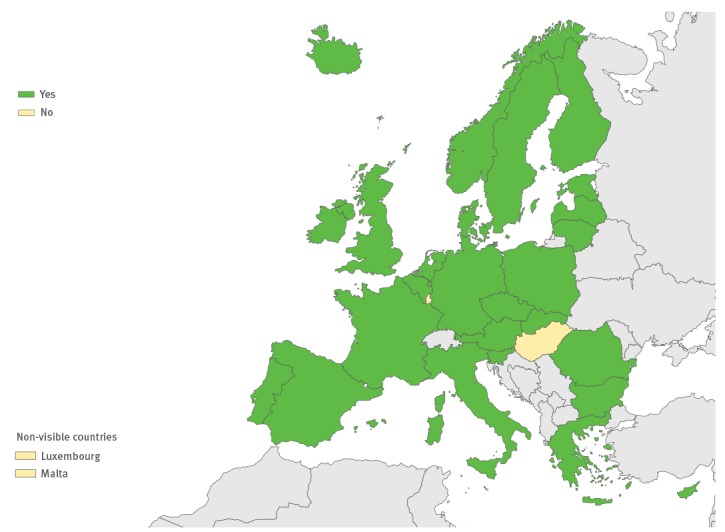
Non-polio enterovirus surveillance systems currently in use in the European Union/European Economic Area countries for enteroviruses, 2016 (n=29 countries)

Twenty-six countries reported that they conducted non-polio EV surveillance based on typing of EVs detected from variety of clinical specimens (Figure 1). Most performed typing of EV-positive samples obtained from individuals with neurological infections, but only half of the countries included respiratory infections, HFMD, myocarditis, haemorrhagic conjunctivitis and post-mortem investigations in their laboratory-based EV surveillance. Countries collected different clinical samples for EV testing from cases of suspected neurological infections; three countries collected only CSF samples, whereas the remaining 23 countries also collected stool and/or respiratory samples in addition to CSF sample. A total of 15 countries took CSF and stool samples, three countries took CSF and respiratory samples, and five countries collected CSF, stool and respiratory samples (data were not available for three countries). Furthermore, eight countries tested all CSF samples for EVs despite the clinical diagnosis and similarly three countries reported that they tested all respiratory samples for EVs.

**Figure 2:**
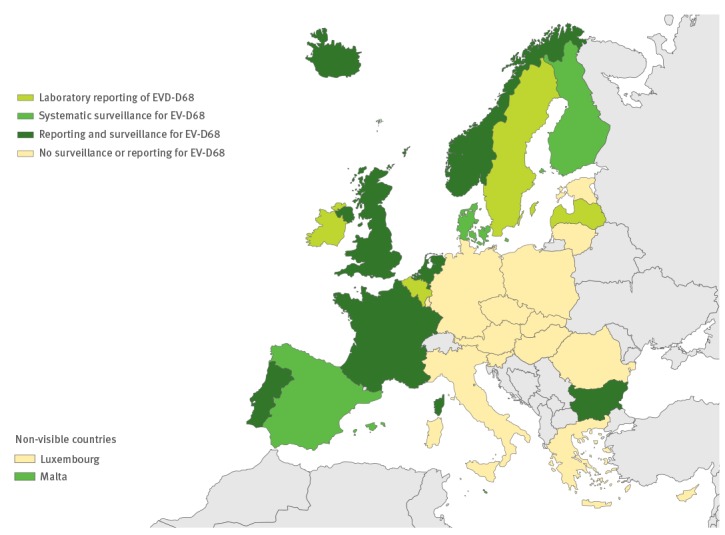
Specific reporting and surveillance systems in use for enterovirus-D68 in the European Union/European Economic Area, 2016 (n=29 countries)

**Figure 3:**
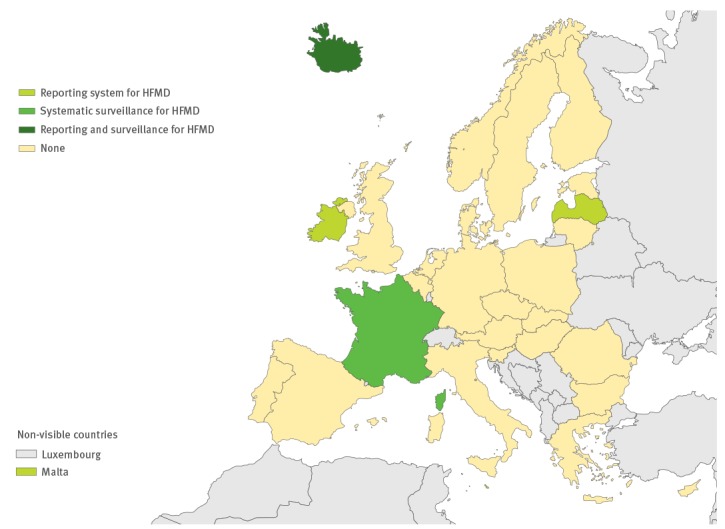
Specific reporting and surveillance systems in use for hand, foot and mouth disease in the European Union/European Economic Area, 2016 (n=29 countries)

Eleven countries had an additional reporting system for EV-D68, and 11 countries have specific surveillance for EV-D68 (Figure 2). This surveillance has been established via sentinel influenza surveillance (n = 7), by typing EV-positive respiratory samples (n = 10) and/or via AFP surveillance (n = 5). Based on the survey response, four countries have established a reporting system for HFMD and two have initiated specific surveillance for it (Figure 3).

EV: enterovirus.

HFMD: hand, foot and mouth disease.

**Figure 4:**
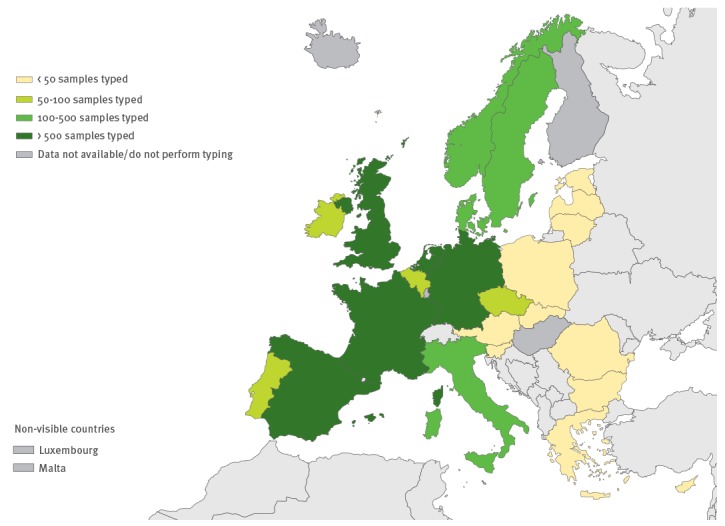
Number of non-polio enterovirus-positive samples typed in European Union/European Economic Area, 2015 (n=29 countries)

As an indicator for surveillance activity, respondents were asked to provide the number of typed EV samples in their country during 2015; over 5,000 EV-positive specimens were reported to have been typed in the EU/EEA countries in 2015 (average per country: 301; range: 0–1,952). The estimated number of typed EV specimens was < 50 in 11 countries (including all countries that typed by neutralisation assay only), whereas six countries successfully typed over 300 EV specimens each in 2015, mostly through sequencing (Figure 4).

### Laboratory capacity

**Figure 5:**
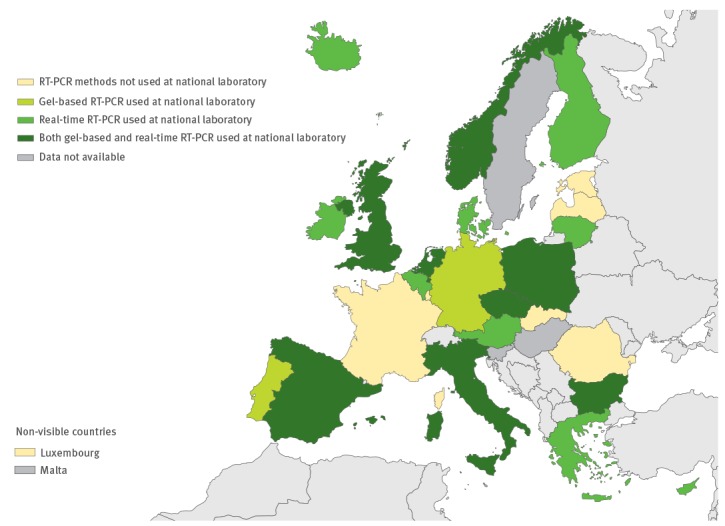
RT-PCR used for primary enterovirus detection in the European Union/European Economic Area, 2016 (n = 29 countries)

**Figure 6:**
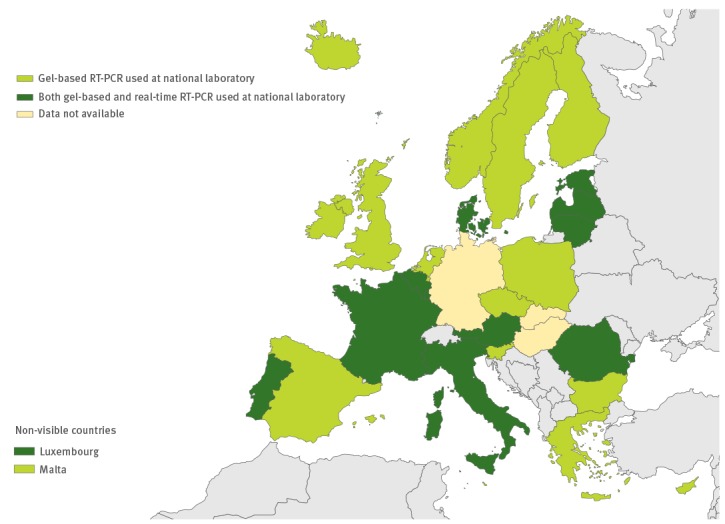
Use of commercial methods in enterovirus diagnostics in the European Union/European Economic Area, 2016 (n = 29 countries)

**Figure 7:**
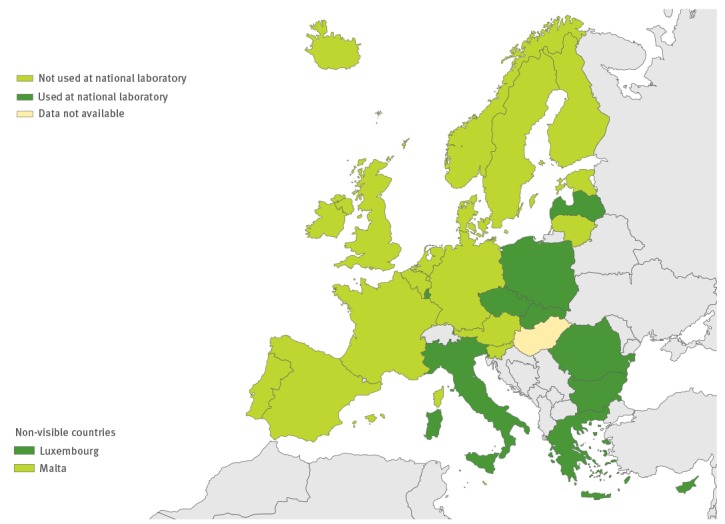
Use of IgG and/or IgM serology in the European Union/European Economic Area, 2016 (n = 29 countries)

**Figure 8:**
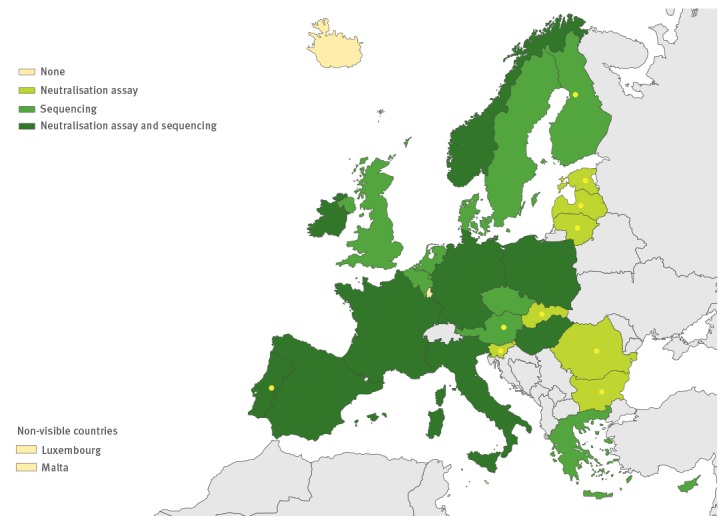
Methods used for enterovirus typing/characterisation in the European Union/European Economic Area, 2016 (n = 29 countries)

A total of 28 countries provided EV testing at the national level, including primary EV testing of clinical specimens in 25 and EV typing in 26 countries (Table, Figures 5,6,7,8). Testing was performed either at the national EV (n = 24), poliovirus (n = 2) or influenza virus (n = 2) laboratory. The reported number of other laboratories performing primary testing varied from one to 89; and in five countries the national laboratory was the only one providing EV testing. Additional non-national laboratories in 11 countries (range 1–11) performed also EV typing. Most national laboratories participated regularly, either every year or biannually, in external quality control programmes for EV detection (n = 25) and characterisation (n = 20).

Yellow spots mark the countries which type culture-positive enterovirus samples only.

### Laboratory methods used

All except one country performed virus isolation at the national level, and 10 countries also performed it at the local level. RT-PCR and/or commercial methods were used for EV detection by all 26 countries performing primary EV testing (Figure 5 and 6). Real-time RT-PCR for EV detection was used in 16 national laboratories. Eight national laboratories performed IgG and/or IgM serology for diagnosing non-polio EVs (Figure 7). Of the 26 countries performing non-polio EV characterisation/typing, 10 characterised only culture-positive EV isolates, whereas the remaining also typed PCR-positive samples. A total of 19 countries reported that they used sequencing-based methods for non-polio EV typing, while neutralisation assay was used by 16 countries. Seven countries in the eastern parts of the EU/EEA relied entirely on neutralisation assay, and did not yet perform non-polio EV typing/characterisation by sequencing (Figure 8).

### Specificity of detection methods used

**Figure 9:**
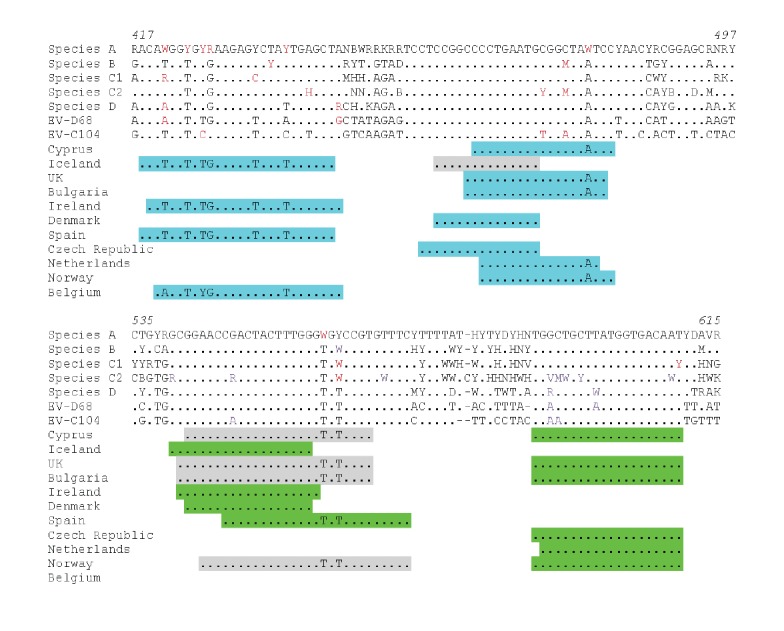
Comparison of primer and probe sequences used in non-polio enterovirus detection RT-PCRs to consensus sequences in various European Union/European Economic Area, 2016 (n=11 countries)

A total of 20 countries use RT-PCR for EV detection; four of them use the WHO-recommended screening primers targeting the VP4 region [26] and the remaining 16 countries use primers targeting the 5’UTR. Primers and probes used for 5’UTR RT-PCR targeted, in general, similarly conserved regions among different EV types (Figure 9; data not available for five countries). Most primers and probes showed good matches to the species A to D EVs, but several mismatches were observed with forward primers targeting the nucleotides between 420 and 440. However, no critical mismatches were observed within the end of the primers or probes used.

EV: enterovirus.

Primers and probe sequences matched to 95% consensus sequences obtained for species A to D enteroviruses and to individual EV-D68 (accession number AY426531) as well as EV-C104 (accession number KC785523) sequences. Two consensus sequences have been created to species C due to the genetic variability in the 5’UTR; species C2 consensus sequence is based on EV-C96, C99, C102, C014, C113, C116, C117 and C118 sequences obtained from GenBank whereas C1 contains all the remaining species C EVs. Sequences from all 106 EV types have been used to create consensus sequences shown in the alignment. Forward primers are shaded blue, and reverse primers green; probes are marked in grey (note only in countries which use real-time RT-PCR). Sequence alignments are numbered on the basis of the CAV16 nucleotide sequence (accession number U05876). For Belgium, reverse primer is outside the region shown.

## Discussion

We provide an overview of national surveillance and detection systems currently used for non-polio EVs within the EU/EEA. Although laboratory-based non-polio EV surveillance is currently in place in most of the countries (26/29), it does not always include neurological infections and the surveillance activity measured as the number of reported EV-positive samples typed in 2015 varied remarkably between countries. It is of concern that 11 countries had typed fewer than 50 EV-positive samples. This is likely to be an insufficient number, taking into account the large variety of circulating EV types [8].

According to the current WHO guidance, an effective EV surveillance system should investigate a ‘significant proportion’ of reported clinical cases, and 80% is given as an example of a significant proportion in that document [26]. A recent study from the United Kingdom showed an incidence of 3.9 per 100,000 for viral meningo-encephalitis based on laboratory-confirmed cases reported in 2013; EVs were shown to be accountable for over half of these cases [32]. If a similar incidence is applied to the EU/EEA population, this would translate to over 10,000 cases of meningoencephalitis caused by EVs annually, and hence at least 8,000 of them should be further investigated with an effective EV surveillance system. Although a total of 7,534 EVs were successfully typed in 2015, data on the proportion of neurological infections among those were not available and will be the subject of future studies.

Systematic reporting and/or surveillance for EV-D68 has not been implemented in the majority of EU/EEA countries; only 11 countries have introduced a surveillance system for EV-D68 infections using existing sentinel surveillance systems, by typing EV-positive respiratory samples or via AFP surveillance (Figure 3). Current sentinel surveillance for respiratory pathogens often includes non-hospitalised patients with either ILI or acute respiratory infection (ARI), and is already in place in the 29 of the 30 EU/EEA countries [33]. Although samples collected via the sentinel ILI and/or ARI surveillance systems have been used for systematic evaluation of the role of EV-D68 infection in Germany, the Netherlands and Canada in 2014 [34-36], it is unlikely that such screening would capture the severe cases of neurological infections associated with EV-D68. Only five cases of AFP associated with EV-D68 have been formally reported in the EU/EEA countries by the end of 2016; two of those were identified in Norway via AFP surveillance [37], whereas one case from France [38] and two cases from Wales [39] were identified via enhanced hospital-based laboratory surveillance established by the European Society for Clinical Virology and ECDC collaborative data collection initiative [20]. Our results show that many countries have chosen more than one system for EV-D68 surveillance, which is important, as the epidemiology of EV-D68 infections is still not fully understood. The previous investigation has suggested that EV-D68 infections might reflect a 2- to 3-year epidemic cycle, as also previously shown for other EV types [13,35]. More data are needed to confirm this epidemiological pattern, and also to demonstrate how often these respiratory infections lead to severe neurological symptoms. Hence, continued careful monitoring and vigilant testing of respiratory samples for EV-D68 and for other non-polio EVs are still needed.

HFMD surveillance in the EU/EEA countries has rarely been implemented in an effective way, with only two countries possessing an established specific surveillance system for patients presenting with this disease. On the other hand, 24 countries with laboratory-based EV surveillance always included aseptic meningitis and other neurological infections in their surveillance system, and also subjected EV-positive CSF samples to further typing. This would mean that although data on uncomplicated HFMD infections are not systematically collected throughout the region, the current non-polio EV surveillance would potentially capture the severe neurological presentations associated with HFMD and/or EV-A71, or any other EV type. Over 80 cases of EV-A71 infections associated with neurological symptoms were recently identified in Spain via the non-polio EV surveillance [40]. However, to optimise laboratory-based EV surveillance including neurological infections, it is important to consider which sample types are tested for EV [21,41]. Collection of stool samples in addition to CSF samples will increase the sensitivity of EV detection in case of EV-A71 infection, whereas respiratory samples are important in view of the sensitive detection of EV-D68 infection [21,41]. According to our survey, only five countries consider inclusion of CSF, respiratory and stool samples for EV testing in case of neurological infection at the national level; this is a result which needs further improvement since testing of stool and respiratory samples substantially enhances the sensitivity of EV-A71 and EV-D68 detection associated with neurological disease.

The use of appropriate testing methodologies is of the greatest importance for any laboratory-based surveillance system. All national laboratories performing primary EV diagnostic used either RT-PCR or commercial methods for EV detection. This should make it easier to introduce more uniform screening of EVs in clinical samples with well-defined sensitivities and specificities on a much greater scale. Laboratories may consider testing all CSF samples for EVs, as is already done in eight countries within the EU/EEA. However, molecular detection of EVs can be challenging due to the genetic diversity of EV types. As we identified a few potential mismatches with primers in terms of EV-D68 and EV-C104 detection, it is important to consider that not all previously published primers (or commercial assays) can detect all EV types. Furthermore, molecular typing of EV-positive samples has also traditionally focused on species B EVs. It is important to note that use of additional primer pairs for typing species A and D EVs does enhance the success rate of molecular typing [8,27,42-44]. A broader understanding of comparability of different assays is also essential, especially in view of emerging viruses with public health importance. Participation in regular external quality control programmes helps laboratories to identify gaps in detection capabilities.

Although direct molecular typing from clinical material has been well-established for EVs [8,42-44], nine countries reported that they only characterised culture-positive EVs. Although data on the cell lines used for EV cultivation were not collected in this survey, it is known that many laboratories focusing primarily on isolation of poliovirus use only L20B and RD cells, based on WHO instructions [45]. CBVs and EV-A71 are known to grow very poorly in these cells, and EV-D68 requires lower incubation temperature than normally applied for EVs [46]. Virus isolation is furthermore known to be less sensitive and much slower than molecular methods, which are easier to implement on the larger scales required for effective EV surveillance [43,46]. Recent changes to poliovirus surveillance algorithms will further mandate the use of molecular methods [47]. Current WHO guidelines state that stool samples should first be cultivated in L20B cells and then isolated polioviruses characterised using poliovirus type-specific real-time PCR instead of traditionally used neutralisation tests. Antibody panels previously used for EV neutralisation essays will no longer be provided by WHO in collaboration with the National Institute for Public Health and the Environment (Bilthoven, the Netherlands) [47]. This means that the antiserum pools used for EV typing will only be available via commercial route in the near future. It is very important to consider what is required for the introduction of a sequencing method for EV surveillance by those seven countries still relying on EV neutralisation assay. Improved capacity for sequencing would be important not only in view of EV surveillance, but also in view of poliovirus eradication and other pathogen surveillance programmes.

### Limitations

As the surveillance and detection systems applied may change from time to time, it is important to note that this study reflects the situation in individual countries as described in 2015 and 2016. This study was mainly based on the EV surveillance and detection activities performed within the national public health institutions; we have not collected information on laboratory capacities and performances at the sub-national level. Furthermore, we have not collected data on research activities in the field of EVs in each EU/EEA country. The potential regional variations in EV surveillance and/or detection activities within the individual EU/EEA countries have not been considered in this study either.

### Conclusions

The recent outbreaks of EV-D68 and EV-A71 infections in Europe emerging over the past 3 years have posed major concerns for local, national and international public health organisations. This is the first time information has been systematically gathered on the variety of existing surveillance systems and laboratory detection methods currently used for non-polio EVs within the EU/EEA countries. This survey highlighted substantial variability in non-polio EV surveillance and identified a clear need to strengthen non-polio EV detection capability and focus on neurological and respiratory infections. National guidance on testing should consider inclusion of respiratory and stool samples in addition to CSF samples for EV identification and characterisation in case of suspected neurological infections. Not only the introduction of molecular methods and sequencing for non-polio enterovirus detection and typing but also systematic data collection and monitoring would improve laboratory response to (re)-emerging EVs linked to severe disease.
